# Poliovirus 2A Protease Triggers a Selective Nucleo-Cytoplasmic Redistribution of Splicing Factors to Regulate Alternative Pre-mRNA Splicing

**DOI:** 10.1371/journal.pone.0073723

**Published:** 2013-09-16

**Authors:** Enrique Álvarez, Alfredo Castelló, Luis Carrasco, José M. Izquierdo

**Affiliations:** 1 Centro de Biología Molecular Severo Ochoa, Consejo Superior de Investigaciones Científicas, Universidad Autónoma de Madrid (CSIC-UAM), Madrid, Spain; 2 European Molecular Biology Laboratory (EMBL), Heidelberg, Germany; International Centre for Genetic Engineering and Biotechnology, Italy

## Abstract

Poliovirus protease 2A (2A^pro^) obstructs host gene expression by reprogramming transcriptional and post-transcriptional regulatory events during infection. Here we demonstrate that expression of 2A^pro^ induces a selective nucleo-cytoplasm translocation of several important RNA binding proteins and splicing factors. Subcellular fractionation studies, together with immunofluorescence microscopy revealed an asymmetric distribution of HuR and TIA1/TIAR in 2A^pro^ expressing cells, which modulates splicing of the human Fas exon 6. Consistent with this result, knockdown of HuR or overexpression of TIA1/TIAR, leads to Fas exon 6 inclusion in 2A^pro^-expressing cells. Therefore, poliovirus 2A^pro^ can target alternative pre-mRNA splicing by regulating protein shuttling between the nucleus and the cytoplasm.

## Introduction

Poliovirus (PV), a member of the Enterovirus genus in the *Picornaviridae* family, contains a single-stranded RNA genome encoding a large polyprotein, which is processed by the viral proteases, 2A^pro^ and 3C^pro^ [1]. These proteases can also cleave host factors and are engaged in the inhibition of host gene expression and also the dismantling of cellular antiviral responses triggered during infection [2]. This can be illustrated by the proteolytic hydrolysis of eukaryotic translation initiation factors 4G (eIF4GI) and eIF4GII, and the poly(A)-binding protein (PABP), which leads to a down-regulation of host cell translation during PV infection [2], [3]. Furthermore, both 2A^pro^ and 3C^pro^ also target various host nuclear proteins, including several transcription factors, which results in an inhibition of cellular RNA synthesis [4]. In addition, during PV infection the specific cleavage of Gemin-3 by 2A^pro^ results in decreased assembly of small nuclear ribonucleoprotein (snRNP) U1 [5]. Using transfected cells and in vitro splicing assays, we recently reported that 2A^pro^ activity controls alternative splicing of pre-mRNAs [6]. However, the mechanism by which 2A^pro^ could modulate splicing events is unknown. PV 2A^pro^ has been shown to target the nuclear pore structure by cleaving several nucleoporins (Nups), including Nup62, Nup98, and Nup153 [7], [8], [9], [10]. This event disrupts protein and RNA trafficking between the nucleus and cytoplasm and, as a consequence, proteins normally retained in the nucleus redistribute to the cytoplasm in infected cells, where they are available to participate in viral protein synthesis and RNA replication [11], [12]. This nuclear to cytoplasm translocation may also impact host gene expression by modulating post-transcriptional regulatory events involving pre-mRNA splicing, mRNA transport and mRNA stability and/or translation. We report here that 2A^pro^-mediated nucleo-cytoplasmic distribution of splicing factors is a potential mechanism to regulate alternative splicing.

## Materials and Methods

### Cell Cultures

Huh7-T7 cells were supplied by R. Bartenschlager (University of Heidelberg, Germany) and grown as described [13].

### Plasmids and Transfections

Expression plasmids pTM1-2A, pTM1-2AM and pTM1-3C, which encode PV 2A^pro^, an inactive 2A^pro^ mutant, and PV 3C^pro^, respectively, have been described previously by us [8]. Plasmids encoding PV 2A^pro^ or 2AM fused to the influenza haemmaglutinin epitope (HA) were generated by PCR amplification using the oligonucleotides OLI-2A-forward: 5′-CCATATGCTCATTGGCCATGGATTC-3′ and OLI-2AHA-reverse: 5′-CGCCGGCGTCGACTATTAAGCGTAATCTGGAACATCG TATGGGTATTGTTCCATCGCTTCTTCTTCGTAGG-3′. The resulting PCR products were digested with NcoI and SalI and cloned into the pTM1-2A or pTM1-2AM plasmids to generate pTM1-2A-HA and pTM1-2AM-HA. Plasmid pRLuc31, containing the PV replicon, was provided by R. Andino [14]. The wild-type Fas minigene and its derivatives, together with protocols for PCR analysis of alternatively spliced RNA products were described previously [15], [16], [17]. The GFP-HuR plasmid, together with protocols for HuR knockdown, were described previously [18]. Plasmids pMS2, pMS2-TIA1 and pMS2-TIAR were previously reported [16]. Transfections were performed with Lipofectamine 2000 (Invitrogen).

### Subcellular Fractionation, Western Blot Analyses and Confocal Microscopy

Transfected Huh7-T7 cells were collected at 4 hours post transfection (hpt), fractionated and analyzed as described [8]. Confocal microscopy was performed as reported [19]. Antibodies to the following proteins were used as reported: PV 2A^pro^ and 3C^pro^, eIF4GI, eIF4GII, Gemin-3, GAPDH, Ref1, TIA1, TIAR, HuR, U2AF65, U2AF35, PTB and hnRNPA1 [6]. To detect 2A-HA and 2AM-HA by immunoblot and microscopy, mouse monoclonal (Covance) and rabbit polyclonal (Sigma) antibodies directed to the HA epitope respectively, were used at 1∶1000 dilution.

## Results and Discussion

### Alternative Splicing of Fas Exon 6 is Regulated by PV 2A^pro^

**Figure 1:**
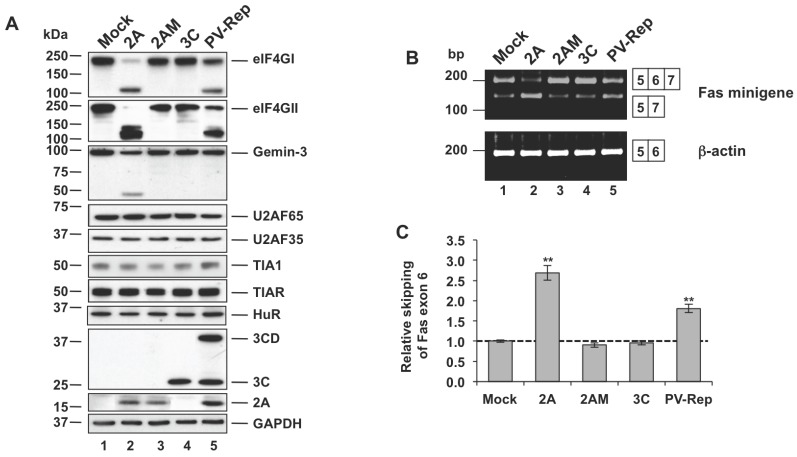
Expression of 2A^pro^ in Huh7-T7 cells. (A) Integrity of splicing factors in 2A^pro^-expressing Huh7-T7 cells. At 4 hpt, the integrity of the indicated proteins was analyzed by immunoblotting with the respective antibodies. An antibody to GAPDH served as a loading control. (B) RT-PCR analysis of alternatively spliced products derived from Fas minigene (*upper*) or ß–actin (*bottom*) genes. Alternatively spliced isoforms are indicated to the right. (C) Intensity of the bands from Fas minigene gel was calculated by densitometry and the values of ratios between 5–7 and 5–6–7 amplification products were expressed as mean ± s.d. (n = 2; **P<0.01 by Student’s *t*-test). The dotted line indicates the ratio of the control sample.

Our previous data showed that over expression of 2A^pro^ in HeLa cells abolished both constitutive and alternative splicing of Fas and FGFR2 pre-mRNAs, and promoted the exclusion of Fas exon 6 in a time and dose-dependent manner [6]. To begin to explore this mechanism we first assayed the integrity of nuclear factors involved in mRNA splicing after expression of 2A^pro^. Consistent with results using different cell lines [5], [8], we found that plasmid-expressed 2A^pro^ cleaved eIF4GI and eIF4GII proteins completely, and Gemin-3 partially, in Huh-7 cells (Figure 1A, lane 2). These results were recapitulated with 2A^pro^ expressed using a poliovirus replicon (PV-Rep) (Figure 1A, lane 5). As expected, expression of a 2A^pro^ point mutant (2AM) lacking proteolytic activity, or wild type 3C^pro^ (3C), failed to cleave these host factors (Figure 1A, compare lane 2 with lanes 3 and 4), [8]. Importantly, additional RNA-binding proteins, U2AF65, U2AF35, TIA1, TIAR and HuR, which can function as general and auxiliary splicing factors, were not cleaved by 2A^pro^ (Figure 1A). These findings collectively illustrate the selectivity of 2A^pro^ proteolytic activity in Huh7-T7 cells.

To study the relationship between 2A^pro^ activity and alternative splicing we chose the well-characterized model of Fas exon 6 splicing, in which exon 6 is differentially included or skipped depending on the repertoire of RNA-binding proteins (RBPs) present [15], [16]. This splicing event is biologically significant because it can regulate the sensitivity of Fas-expressing cells to Fas-induced apoptosis. RT-PCR analysis of co-transfected Fas minigene expression demonstrated an increased frequency of exon 6 skipping, upon 2A^pro^ expression in Huh7-T7 cells (plasmid and PV replicon, Figure 1B and 1C), which was consistent with previous results [6]. As before, expression of 2AM and 3C failed to modify Fas exon 6 minigene splicing (Figure 1B and 1C). The expression pattern of ß-actin, used as a control, was not affected. These findings suggest that 2A^pro^ promotes changes in the alternative splicing of Fas receptor.

### U-rich Sequence on Exon 6 (URE6) is Essential for Fas Exon 6 Skipping in PV 2A^pro^-expressing Cells

**Figure 2:**
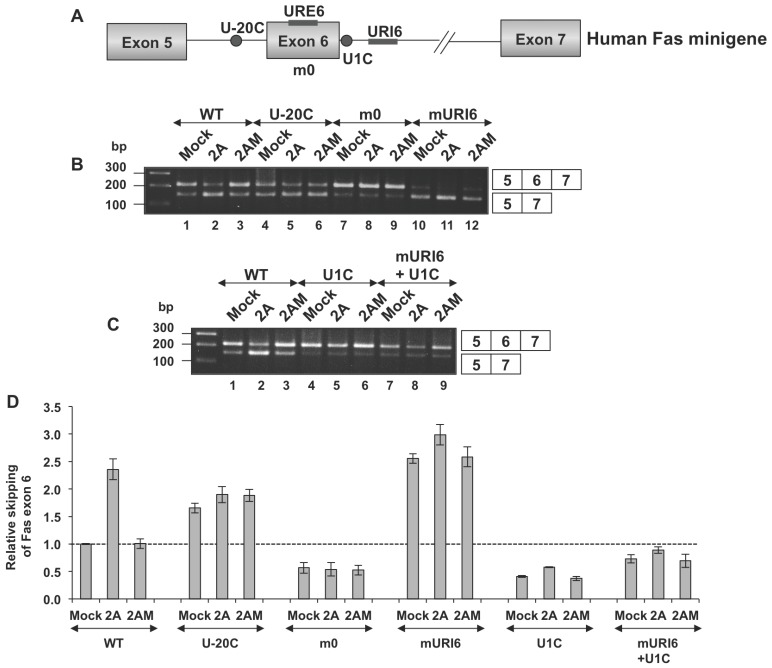
The URE6 sequence mediates skipping of Fas exon 6 in 2A^pro^-expressing cells. (A) Schematic diagram of the human Fas minigene (Fas genomic sequence from exon 5–7) and mutations used (Izquierdo, 2010). (B and C) Huh7-T7 cells were transfected with, pTM1-2A or pTM1-2AM or empty (mock) plasmid and analyzed as in Figure 1B. (D) Intensity of the bands from panels B and C was calculated by densitometry and the values of ratios between 5–7 and 5–6–7 amplification products were expressed as mean ± s.d. (n = 2). The dotted line indicates the ratio of the control sample.

In order to define the regulatory elements necessary for 2A^pro^-dependent splicing alterations, we next utilized mutated Fas minigene reporters in transient transfection assays (Figure 2A) [16]. In agreement with these studies, mutation U-20C (Fas with a uridine to cytidine substitution 20 nucleotides upstream from the 3′ splice site of intron 5) resulted in a natural skipping of exon 6, and no additive effect was observed after expression of 2A^pro^ (Figure 2B, lanes 1–2 vs lanes 4–5 and Figure 2D). Interestingly, variant m0, with replacement of a uridine-rich sequence on exon 6 (URE6), was refractory to 2A^pro^, and exon 6 was prominently included in all cases (Figure 2B, compare lanes 1–6 with lanes 7–8 and Figure 2D). This result indicated that the URE6 sequence was important for exclusion of exon 6 in 2A^pro^-expressing cells. Furthermore, when sites for the RNA-binding proteins TIA1/TIAR in the U-rich sequence on Fas intron 6 (URI6) were replaced by an unrelated sequence, increased skipping of exon 6 was observed in mock transfected cells which was enhanced by 2A^pro^ expression (Figure 2B, compare lanes 10 and 12 with 11 and Figure 2D). These findings indicate that TIA1/TIAR binding regions were important for exon 6 definition [16], [17], and substitution of these sequences did not suffice to prevent 2A^pro^-mediated splicing defects. However, the mutation U1C in the minigene (positions −2 and −3 from 5′ splice site of exon were mutated to A and C, respectively, and positions 7 and 8 of intron 6 were mutated to A and T, respectively) promoted inclusion of exon 6, and this was refractory to 2A^pro^ activity (Figure 2C, lanes 1–3 vs 4–6 and Figure 2D). Similarly, splicing of the minigene combining both mURI6 and U1C mutations was insensitive to 2A^pro^, suggesting that the 2A-dependent splicing effects can be counteracted by increasing the strength of Fas intron 6 5′ splice site (Figure 2C, lanes 1–3 vs 7–9 and Figure 2D). Taken together, these findings indicate a strong dependence of 2A splicing regulation on the U-rich sequence of Fas exon 6 (URE6), and the strength of the 5′ splice site of Fas intron 6.

### PV 2A^pro^ Selectively Alters the Subcellular Distribution of Splicing Factors

**Figure 3:**
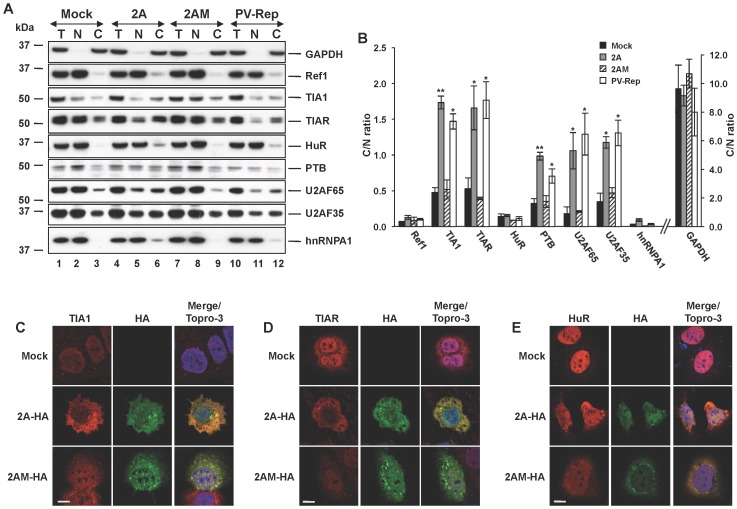
Expression of 2A^pro^ provokes nuclear-cytoplasmic redistribution of splicing factors. (A) Huh7-T7 cells were transfected with pTM1-2A, pTM1-2AM, PV-Rep or empty plasmid. Subcellular distributions of the indicated proteins were analyzed by immunoblot using the respective antibodies. *T* refers to total, *N* refers to nuclear, and *C* refers to cytoplasmic fractions. GAPDH and Ref1 were used as controls for cytoplasmic and nuclear location respectively. (B) The densitometry of the bands was used to calculate the cytoplasm/nucleus ratio for each protein shown. Data are mean ± s.d. (n = 3; *P<0.05; **P<0.01 by Student’s *t*-test). (C-E) Immunofluorescence staining of TIA1, TIAR and HuR proteins in Huh-7-T7 cells. Cells transfected as in (A) were immunolabelled with the indicated antibodies. Merge/Topro-3 refers to simultaneous visualization of images. Scale bars: 10 µm.

The URE6 sequence of human Fas pre-mRNA is a significant *cis*-acting regulating element of Fas alternative splicing, which recruits *trans*-acting factors such as PTB/hnRNPI [16] and/or HuR/ELAVL1/HuA [18] to modulate exon 6 skipping/inclusion. As these splicing factors were not cleaved by 2A^pro^ in our experimental system (Figure 3A), we thought it possible that 2A^pro^ activity may selectively alter the subcellular distribution/localization of these factors. To test this possibility, nuclear and cytoplasmic fractions were obtained from mock-transfected cells, or cells expressing 2A^pro^, or 2AM. Interestingly, immunoblot analysis showed that the regulators of Fas splicing: TIA1, TIAR and PTB together with the essential splicing factors U2AF35 and U2AF65 were all partially redistributed to the cytoplasm in cells expressing 2A^pro^, whereas HuR and hnRNPA1 proteins were retained largely in the nucleus under these conditions (Figure 3A). Analysis of the cytoplasm/nuclear ratio confirmed this significant redistribution of the affected proteins (Figure 3B). Similar results were obtained when the PV-rep was used (Figure 3A, B), whereas expression of the 2AM point mutant failed to promote redistribution of the splicing factors (Figure 3A). As a complimentary approach, we next assessed the subcellular localization of RBPs by confocal microscopy. As antibodies to the 2A^pro^ work poorly for immunofluorescence we first tagged the proteases with an HA-epitope. Immunoblot analysis showed that HA-tagged 2A^pro^ (2A-HA) and HA-tagged 2AM (2AM-HA) had similar expression levels to the untagged proteins in transfected cells, and proteolytic activity against eIF4GI and eIF4GII substrates was also comparable (Figure S1A). As expected, confocal analysis revealed that both 2A-HA and 2AM-HA exhibited a similar localization in the nucleus and cytoplasm of transfected cells (Figure S1B). In agreement with the immunoblot analysis (Figure 3A), inspection of confocal images revealed a redistribution of TIA1 (Figure 3C) and TIAR (Figure 3D), but not HuR (Figure 3E), to the cytoplasm in cells transfected with 2A-HA, which was significantly less apparent in 2AM-HA-expressing cells. Similar expected results were noted for U2AF65, U2AF35 and hnRNP-A1 (Figure S2A–C) when compared to the localization marker protein Ref1 (Figure S2D). Collectively, these results suggest that the protease activity of 2A^pro^ can specifically promote the nucleo-cytoplasmic shuttling of several splicing factors and RNA-binding proteins to tailor a selective gradient of these multifunctional regulators to both sides of the nuclear membranes. This, in turn, may modulate post-transcriptional gene expression of the host cell.

### Overexpression of TIA1 and TIAR Promotes Fas Exon 6 Inclusion in PV 2A^pro^-expressing Cells

**Figure 4:**
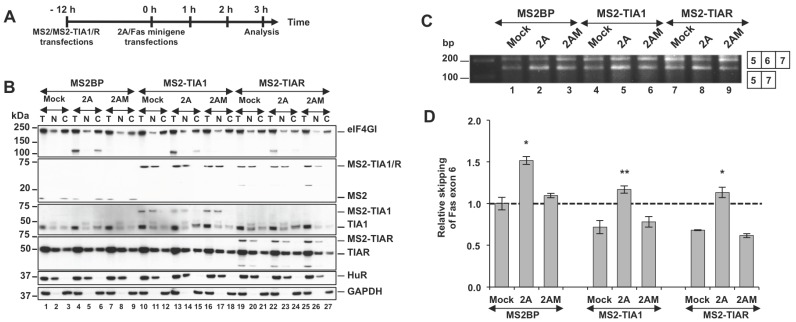
TIA1 and TIAR overexpression promotes Fas exon 6 inclusion in 2A^pro^-expressing cells. (A) Workflow: Huh7-T7 cells were transfected with plasmids expressing MS2BP, MS2-TIA1 or MS2-TIAR. At 12 hpt the cells were co-transfected with Fas minigene and pTM1-2A, 2AM or empty pTM1 plasmid. Cells were analyzed at 3 hpt. (B) Cells from (A) were fractionated as described above and immunoblot analysis using the antibodies indicated was performed (from top to bottom: eIF4GI, MS2BP, TIA1, TIAR, HuR and GAPDH). (C) RT-PCR analysis of alternatively spliced products from cells processed in (B). (D) Intensity of the bands was calculated by densitometry and the values of ratios between 5–7 and 5–6–7 amplification products were expressed as mean ± s.d. (n = 3; *P<0.05; **P<0.01 by Student’s *t*-test). The dotted line indicates the ratio of the control sample.

It is known that the HuR protein serves as a repressor, which promotes Fas exon 6 exclusion [17], [18]. Given this information, the above results indicate that a nuclear imbalance of HuR protein with respect to TIA1 and TIAR protein levels might mediate Fas exon 6 skipping in 2A^pro^-expressing cells. To explore this idea, we applied the experimental strategy outlined (Figure 4A) to overexpress tagged MS2-TIA1 and MS2-TIAR proteins in Huh7-T7 cells. Interestingly, both proteins showed a predominantly nuclear localization, even in 2A-expressing cells (Figure 4B), indicating that the nuclear localization signal present in these constructs is enough to retain these proteins in the nucleus. Under these conditions, an increase of Fas exon 6 inclusion was observed in cells expressing MS2-TIA1 or MS2-TIAR, compared with cells expressing control MS2BP (Figure 4C, compare lanes 4–6 and 7–9, respectively, with 1–3), in a reproducible manner (Figure 4D) reinforcing the idea that TIA/HuR imbalance modulates Fas alternative splicing in 2A^pro^-expressing cells.

### Depletion of HuR Promotes Fas Exon 6 Inclusion in PV 2A^pro^-expressing Cells

**Figure 5:**
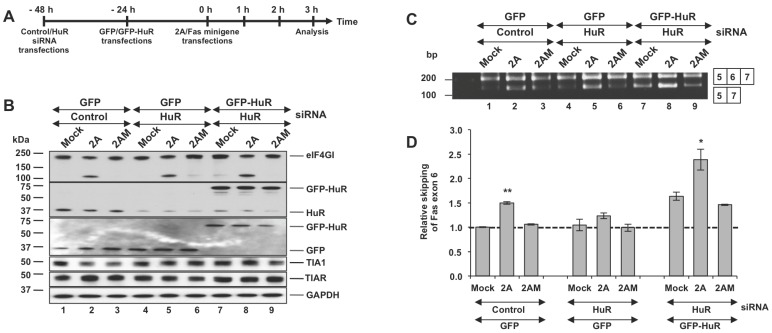
HuR knockdown promotes Fas exon 6 inclusion in 2A^pro^-expressing cells. (A) Workflow: Huh7-T7 cells were transfected with control or HuR siRNA. At 24 hpt the cells were transfected with plasmids expressing GFP or GFP-HuR. Next day the cells were co-transfected with Fas minigene and pTM1-2A, 2AM or empty pTM1 plasmid. Cells were processed for analysis at 3 hpt. (B) Immunoblot analysis from cells processed in (A) using the indicated antibodies (from top to bottom: eIF4GI, HuR, GFP, TIA1, TIAR and GAPDH). (C) RT-PCR analysis of alternatively spliced products from cells processed in (A). (D) Intensity of the bands was calculated by densitometry and the values of ratios between 5–7 and 5–6–7 amplification products were expressed as mean ± s.d. (n = 3; *P<0.05; **P<0.01 by Student’s *t*-test). The dotted line indicates the ratio of the control sample.

To further test the hypothesis that an imbalance between HuR and TIA1/R, induced by 2A^pro^, could modulate Fas alternative splicing we next performed the reciprocal experiment (outlined in Figure 5A) to generate a loss- or gain-of-function of HuR. HuR is an RBP predominantly localized in the nucleus of Huh7-T7 cells (>90%), with nucleolar exclusion; whereas TIA1 and TIAR show a characteristic nuclear-cytoplasmic localization pattern (Figure 3C–E). Nevertheless, HuR and endogenous TIA proteins are antagonistic in their actions related to the alternative splicing of Fas exon 6 [15], [16]. Compared to control siRNA cells, knockdown of HuR in Fas minigene co-transfected Huh7-T7 cells using an siRNA targeted to the 3′UTR, resulted in an 80–90% reduction in steady-state levels of HuR expression (Figure 5B) and, at the same time, led to increased levels of Fas exon 6 inclusion (Figure 5C, lane 1–3 vs lane 4–6) in a reproducible manner (Figure 5D). To exclude off-target effects of the siRNA, a GFP-tagged HuR expression plasmid, which was resistant to the 3′UTR-specific siRNA (Izquierdo, 2008; Izquierdo, 2010) was used under the same experimental conditions. [17], [18]. GFP-HuR was located mainly in the nucleus, even in 2A-expressing cells, whereas GFP showed a combined nucleo-cytoplasmic distribution (Figure S3A, B). As anticipated, expression of GFP-HuR rescued the exon exclusion phenotype despite knockdown of endogenous HuR (Figure 5C, compare lanes 1–6 with 7–9 and Figure 5D). Given that PV 2A also relocalizes PTB, another repressor of Fas exon 6 inclusion [16], to the cytoplasm (Figure 3A) these results strongly suggest that the HuR retained in the nucleus can promote Fas exon 6 skipping in 2A^pro^-expressing cells. Overall, our results support the notion that the selective re-localization of splicing factors from the nucleus to the cytoplasm in 2A-expressing cells, can account for the effect of 2A protease on human Fas exon 6 splicing.

### PV 2A^pro^ Promotes the Nucleo-cytoplasmic Re-distribution of Splicing Factors and RNA-binding Proteins: Implications for PV life-cycle

**Figure 6:**
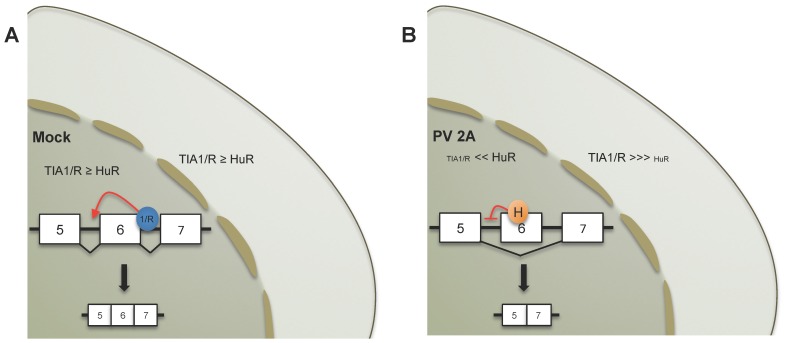
Model for human Fas exon 6 splicing. (A) In the mock, TIA1 (1) and TIAR (R) are located mainly in the nucleus. In these conditions, the amount of TIA1/R is slightly higher that amount of HuR. The binding of TIA1 and TIAR to their corresponding binding site localized in Fas intron 6 promotes exon 6 inclusion. (B) In 2A^pro^-expressing Huh7-T7 cells, TIA1 and TIAR are translocated to the cytoplasm. The binding of HuR (H) to its site promotes Fas exon 6 skipping.

Our present work addresses the molecular mechanisms underlying the activity of 2A^pro^ and its regulation of Fas exon 6 splicing. This is the first report to demonstrate that this splicing event could be regulated by an asymmetric distribution of TIA and HuR proteins between the nuclear and cytoplasmic compartments (Figure 6). Consequently, control of alternative splicing in host cells could emerge as an important target to alter gene expression of infected cells. Indeed, an appreciation of splicing regulation may be important to understand how viruses evade the host antiviral response. Previous work [7], [8], [9], [10] has suggested that the selective cleavage of Nups could facilitate the redistribution of RNA-binding proteins between the nucleus and cytoplasm upon 2A^pro^ expression. In this sense, PV 2A^pro^ can direct the relocalization of the cellular splicing factor SRp20 to the cytoplasm where it interacts with poly(C)-binding protein-2 and stimulates PV translation [11], [12]. Our present observations support the idea that PV 2A^pro^ specifically remodel the nucleo-cytoplasmic distribution of a number of RNA-binding proteins which are involved in post-transcriptional regulatory events. The question of why TIA1 and TIAR relocalize to the cytoplasm, whereas HuR remains in the nucleus in cells expressing PV 2A^pro^ could be explained by the differences in the nuclear localization signals harbored by these proteins as well as the distinct nuclear import/export pathways followed by TIA1/TIAR and HuR [20]. In this sense, it has been described that import/export of TIA1 and TIAR to/from the nucleus significantly differ from that documented for HuR [20], [21]. However, further investigations are required to know the mechanism by which PV 2A^pro^ modulates nucleo-cytoplasmic trafficking, which could potentially give insights into differential pathways of protein shuttling between nucleus and cytoplasm.

## Supporting Information

Figure S1 **Expression of HA-tagged PV 2A^pro^ proteins in Huh7-T7 cells.** (A) Huh7-T7 cells were transfected with pTM1-2A and pTM1-2A-HA. As controls, pTM1-2AM, pTM1-2AM-HA or empty plasmid were transfected also. At 4 hpt, samples were analyzed by Western blotting with antibodies raised to different host proteins as indicated to the right. Molecular mass markers (kDa) for protein are indicated to the left. (B) Distribution of HA-tagged PV 2A^pro^ in Huh-7-T7 cells. Cells were transfected with pTM1-2A-HA or pTM1-2AM-HA or with an empty plasmid as a control. At 4 hpt, cells were fixed and indirect immunofluorescence was carried out using an antibody to HA. Samples were visualized with a confocal microscope. Merge shows the simultaneous α-HA, and Topro-3 to label the nucleus. Scale bar: 10 µm.
(TIFF)

Figure S2 **Subcellular localization of splicing factors.** Huh-7-T7 cells were transfected with pTM1-2A-HA or pTM1-2AM-HA or with an empty plasmid as a control. At 4 hpt, cells were fixed and indirect immunofluorescence was carried out using antibodies to U2AF65 (A), U2AF35 (B), hnRNPA1 (C) or Ref1 (D). Localization of 2A-HA and 2AM-HA were analysed as described above. All samples were visualized with a confocal microscope. Merge shows the simultaneous visualization of the splicing factor, α-HA, and Topro-3 to label de nucleus. Scale bars: 10 µm.
(TIFF)

Figure S3 **Subcellular localization of GFP-HuR.** Huh7-T7 cells were transfected with a control or HuR siRNA. At 24 hpt the cells were transfected with plasmids expressing GFP-HuR (A) or GFP (B). The next day cells were co-transfected with the Fas minigene and pTM1-2A. Mutant 2AM cells transfected with the empty pTM1 plasmid were used as controls. At 3 hpt from the last transfection cells were fixed and the localization of GPF-HuR (A) or GFP(B) was analysed by confocal microscopy. Localization of TIA-1 was also analysed using a specific antibody. Merge shows the simultaneous visualization of the GPF/GFP-HuR, α-TIA, and Topro-3 to label de nucleus. Scale bars: 10 µm.
(TIF)
