# Supplementary figures and images for: Poliovirus 2A Protease Triggers a Selective Nucleo-Cytoplasmic Redistribution of Splicing Factors to Regulate Alternative Pre-mRNA Splicing
Source: PLoS One. 2013 Sep 16;8(9):e73723. doi: 10.1371/journal.pone.0073723 (PMC3774746; doi:10.1371/journal.pone.0073723)

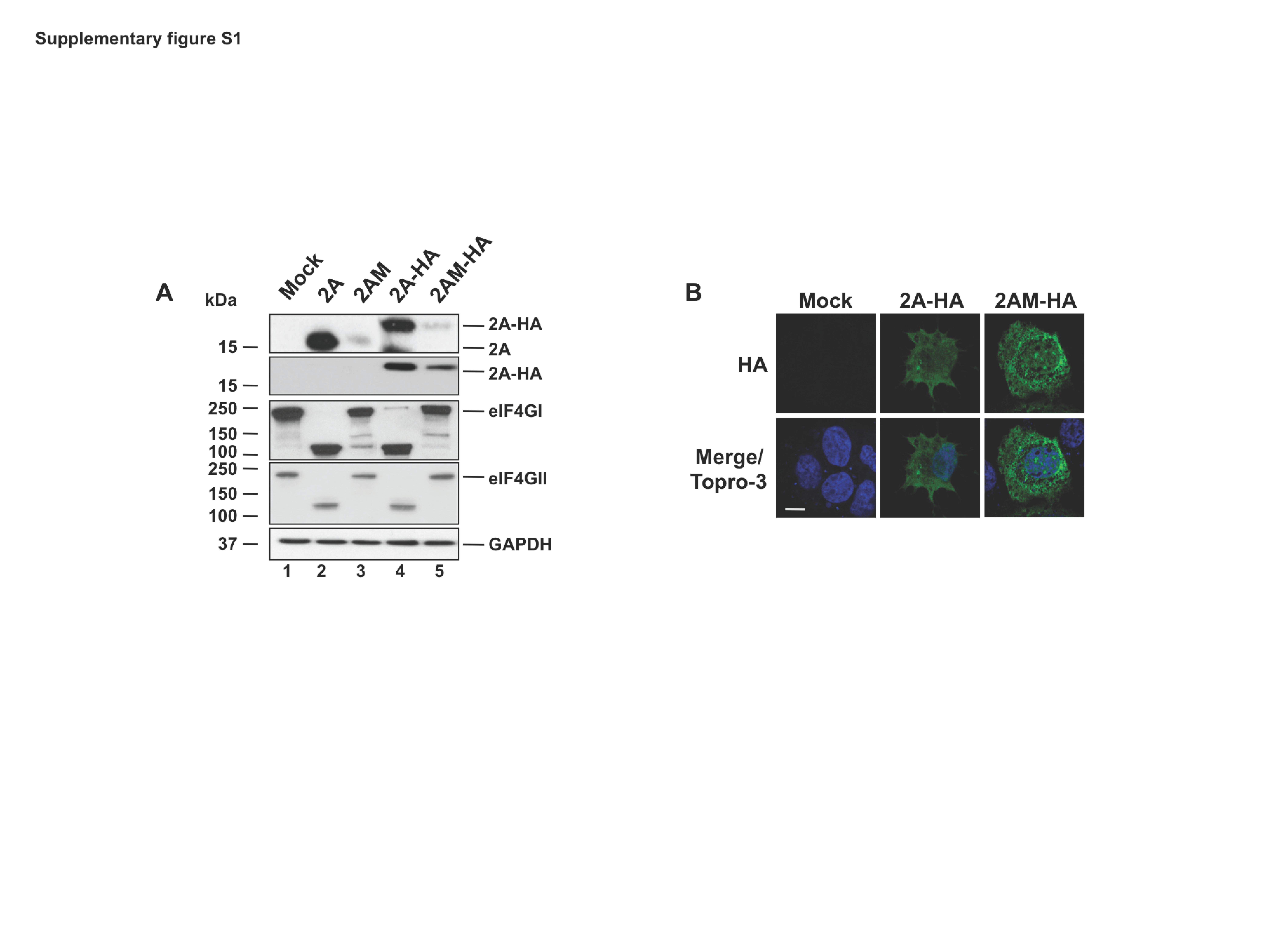

Supplement: Figure S1 — Expression of HA-tagged PV 2Apro proteins in Huh7-T7 cells. (A) Huh7-T7 cells were transfected with pTM1-2A and pTM1-2A-HA. As controls, pTM1-2AM, pTM1-2AM-HA or empty plasmid were transfected also. At 4 hpt, samples were analyzed by Western blotting with antibodies raised to different host proteins as indicated to the right. Molecular mass markers (kDa) for protein are indicated to the left. (B) Distribution of HA-tagged PV 2Apro in Huh-7-T7 cells. Cells were transfected with pTM1-2A-HA or pTM1-2AM-HA or with an empty plasmid as a control. At 4 hpt, cells were fixed and indirect immunofluorescence was carried out using an antibody to HA. Samples were visualized with a confocal microscope. Merge shows the simultaneous α-HA, and Topro-3 to label the nucleus. Scale bar: 10 µm. (TIFF) [file pone.0073723.s001.tiff]

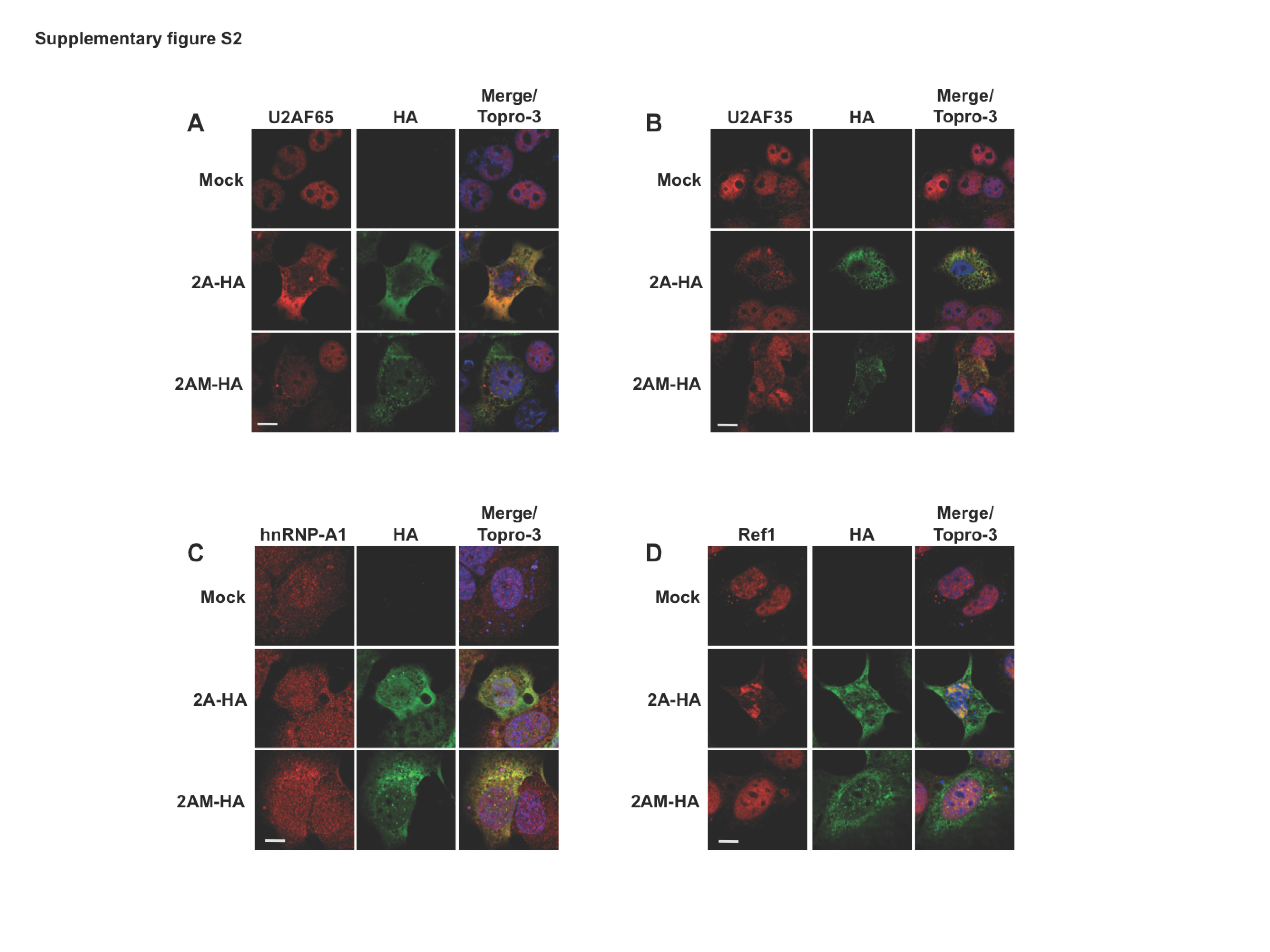

Supplement: Figure S2 — Subcellular localization of splicing factors. Huh-7-T7 cells were transfected with pTM1-2A-HA or pTM1-2AM-HA or with an empty plasmid as a control. At 4 hpt, cells were fixed and indirect immunofluorescence was carried out using antibodies to U2AF65 (A), U2AF35 (B), hnRNPA1 (C) or Ref1 (D). Localization of 2A-HA and 2AM-HA were analysed as described above. All samples were visualized with a confocal microscope. Merge shows the simultaneous visualization of the splicing factor, α-HA, and Topro-3 to label de nucleus. Scale bars: 10 µm. (TIFF) [file pone.0073723.s002.tiff]

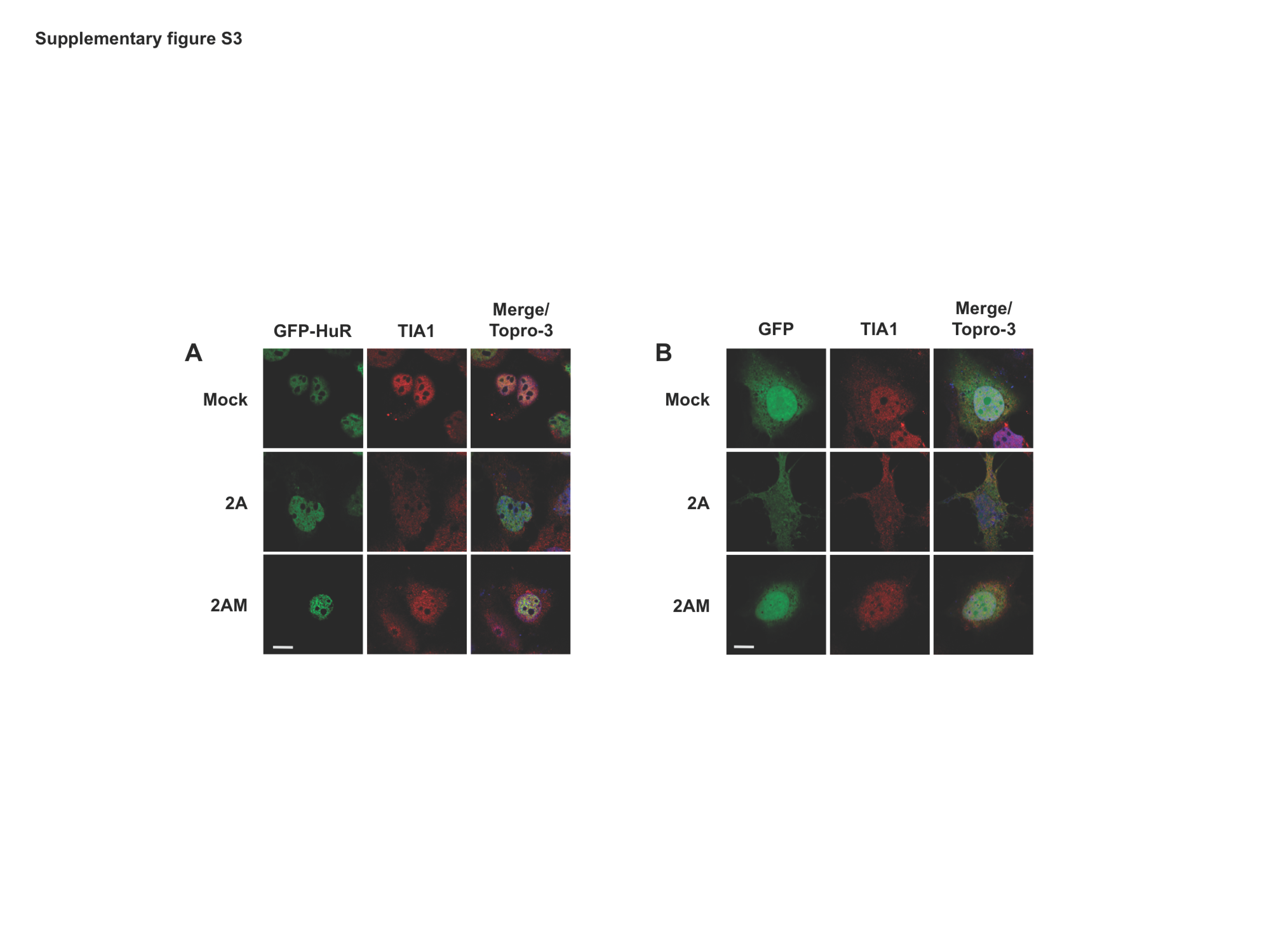

Supplement: Figure S3 — Subcellular localization of GFP-HuR. Huh7-T7 cells were transfected with a control or HuR siRNA. At 24 hpt the cells were transfected with plasmids expressing GFP-HuR (A) or GFP (B). The next day cells were co-transfected with the Fas minigene and pTM1-2A. Mutant 2AM cells transfected with the empty pTM1 plasmid were used as controls. At 3 hpt from the last transfection cells were fixed and the localization of GPF-HuR (A) or GFP(B) was analysed by confocal microscopy. Localization of TIA-1 was also analysed using a specific antibody. Merge shows the simultaneous visualization of the GPF/GFP-HuR, α-TIA, and Topro-3 to label de nucleus. Scale bars: 10 µm. (TIF) [file pone.0073723.s003.tif]
